# Effects of Solid–Solution Temperature on Microstructures and Mechanical Properties of 2200 MPa Grade Secondary Hardening Steel

**DOI:** 10.3390/ma18092126

**Published:** 2025-05-06

**Authors:** Cheng Yang, Yong Li, Shun Han, Xuedong Pang, Ruming Geng, Xinyang Li, Chunxu Wang

**Affiliations:** 1 Institute of Special Steels, Central Iron and Steel Research Institute Co., Ltd., Beijing 100081, China; yc15599035705@163.com (C.Y.); hanshunfa@126.com (S.H.); gengruming@nercast.com (R.G.); lixinyang@nercast.com (X.L.); wangchunxu@nercast.com (C.W.); 2 Technology Center, Fushun Special Steel Co., Ltd., Fushun 113001, China; xuedong0610@163.com

**Keywords:** ultra–high–strength steel, solid–solution, microstructure, primary carbide

## Abstract

With the increasing demands for mechanical properties of ultra–high–strength steels (UHSSs), enhancing their strength and obtaining an excellent strength–toughness matching have received widespread attention. In this paper, the influence of microstructure and primary carbides on the mechanical properties of 2200 MPa ultra–high–strength steel was studied by treating it at different solid–solution temperatures. The mechanical properties of the experimental steel following aging demonstrated a non–monotonic dependence on solid–solution temperature, manifested as an initial increase followed by a gradual decline in both strength and toughness. Microstructural evolution analysis reveals that elevated solid–solution temperatures induce coarsening of prior austenite and martensite grains in the steel, thereby promoting toughness enhancement. Concurrently, primary carbides progressively dissolve into the matrix with increasing solid–solution temperature, generating a supersaturated solid–solution that facilitates M_2_C carbide precipitation during aging, ultimately leading to strength improvement in the experimental steel. An exceptional combination of strength, ductility, and toughness with an ultimate tensile strength of 2142 MPa, yield strength of 1830 MPa, elongation of 12.5%, and Charpy U–notch impact energy of 60.5 J was obtained when the experimental steel was solid–solution treated at 910 °C.

## 1. Introduction

With an increasing demand for high–performance structural materials applied in military aircraft, aerospace, shipbuilding, and power generation industries, the research and development of ultra–high–strength steels (UHSS) has been significantly accelerated to achieve a superior strength–toughness balance. UHSS represents an advanced alloy steel developed from conventional alloy structural steels. This engineered material exhibits an optimal combination of exceptional strength and fracture toughness, achieving specific strength properties critical for weight–sensitive applications. Improving strength to maximum specific strength is an ongoing development goal for UHSS, especially for the aerospace industry [1,2]. UHSS is systematically classified into three types based on its total alloy content, including low–alloy UHSS (<5 wt% alloying elements), medium–alloy UHSS (5–10 wt%), and high–alloy UHSS (>10 wt%) [3]. Low–alloy and medium–alloy UHSSs have low alloying element content and a simple production process, but their inadequate strength and toughness coordination, lack of fracture toughness, and poor stress corrosion resistance limit their application in high–performance aerospace components [4,5]. High–alloy UHSS includes martensitic aging steel, secondary hardening UHSS, and ultra–high strength stainless steel. Under the same strength, high–alloy ultra–high strength steel has the best combination of strength, toughness, and fatigue performance [1,2,6,7,8,9].

The heat treatment process for secondary hardening UHSS usually includes solid–solution, cryogenic, and aging. During the aging process, semi–coherent M_2_C carbides composed of alloying elements including Cr, Mo, W, etc., were formed to pin dislocations, thus causing the secondary hardening effect and providing a strength enhancement of 300–500 MPa [10,11,12,13,14,15,16].

However, the precipitation of M_2_C carbides is also greatly affected by the solid–solution process [17,18]. The purpose of solid–solution treatment is to promote the re–dissolution of large–sized carbides formed during the forging process and make the alloying elements in the matrix supersaturated before aging, thereby promoting the full precipitation of M_2_C–carbides during the aging process [19,20,21,22]. Therefore, a low solid–solution temperature will not only reduce the precipitation amount during the aging process but also leave behind large–sized carbides in the forged state, which will detrimentally affect the material’s performance.

Furthermore, the solid–solution temperature directly affects the microstructures of the martensite matrix, as excessively high solid–solution temperatures lead to coarse austenite grains, further affecting the lath width of the martensite matrix, the geometrically necessary dislocation density, and the evolution of the grain boundary character distribution [23,24,25]. Wang et al. [26] studied the effect of solid–solution temperature on AIR0509 steel, indicating that the size of primary austenite grain (PAG) and martensite microstructures coarsened with increasing solid–solution temperature, especially resulting in a rapid PAG growth from 26 μm to 80 μm as the temperature exceeded 1000 °C. Bao et al. [27] demonstrated that the geometrically necessary dislocation density (*ρ_GND_*) in martensite decreased with increasing solid–solution temperature, while that in austenite was irregularly distributed, and the austenite stress was easy to concentrate in certain grains.

In the present study, the mechanical properties and microstructure evolution mechanisms of a secondary hardening UHSS at different solid–solution temperatures were analyzed, obtaining an exceptional strength–toughness combination. Combined with thermodynamic calculations, energy–dispersive spectroscopy (EDS), and transmission electron microscopy (TEM) characterization, the primary carbides in the experimental steel were confirmed to be (Mo, Cr)_6_C.

## 2. Materials and Methods

### 2.1. Materials and Heat Treatment

**Figure 1:**
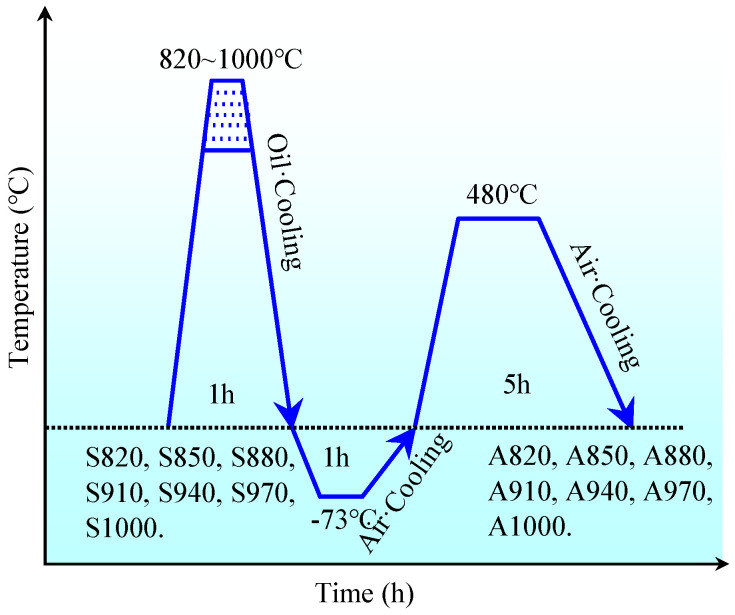
Heat treatment steps and sample classification of experimental steel.

The nominal chemical compositions of experimental steel are listed in Table 1. It was manufactured in a 50 kg vacuum induction furnace using raw materials and cast into ingots. The casted ingots were forged to square billets with a cross–section dimension of 25 mm × 15 mm, in which the reheating temperature was 1200 °C for 6 h and the beginning and finishing forging temperatures were 1180 °C and 800 °C, respectively. To reduce internal stress, the annealing process was then carried out at 680 °C for 5 h. After that, the experimental steels were treated through oil quenching, cryogenic treatment, and aging. To investigate the effects of solution temperature on microstructure and mechanical properties, specimens were solid–solution treated at 820 °C, 850 °C, 880 °C, 910 °C, 940 °C, 970 °C, and 1000 °C for 1 h using a TSX–8–14 high–temperature heat treatment furnace (rated power: 8 kW, rated voltage: 380 V, rated temperature: 1250 °C) to enhance microstructural homogeneity and ensure complete dissolution of coarse carbides, followed by oil quenching to room temperature. After solid–solution treatment, direct testing and subsequent aging were conducted separately. All aged specimens were maintained at −73 °C for 1 h in a WD8–0.4 FB ultra–low temperature chamber to facilitate complete austenite–to–martensite transformation, then air–cooled to ambient temperature. Subsequent aging was performed at 480 °C for 5 h using a TSX–8–12 medium–temperature furnace (rated power: 8 kW, rated voltage: 380 V, rated temperature: 1050 °C) to precipitate supersaturated alloying elements as carbides, followed by air cooling to room temperature. Detailed thermal processing procedures, temperature profiles, and specimen classification are presented in Figure 1. Specimens without aging treatment were designated as S820, S850, S880, S910, S940, S970, and S1000 according to their solution temperatures, while aged counterparts were labeled A820, A850, A880, A910, A940, A970, and A1000.

### 2.2. Thermodynamic Calculation and Solid–Solution Temperature

**Figure 2:**
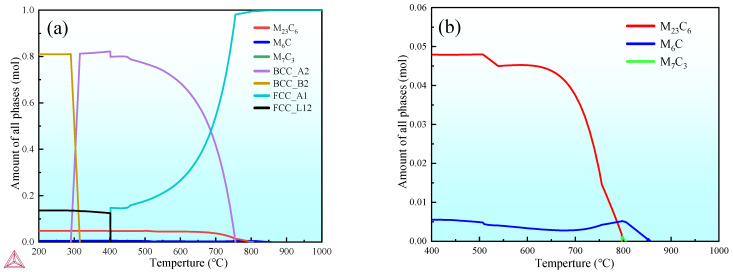
Thermodynamic calculation. (**a**,**b**) Phase composition at equilibrium temperature.

The formation of M_6_C, MC, and M_2_C types of carbides during heat treatment has a significant impact on the properties of the steel, so it is of great importance to study the dissolution process and precipitation behavior of carbides in secondary hardening steels at different solid–solution temperatures [7]. Figure 2 shows the thermodynamic results calculated using Thermo–Calc software from the TCFE7 database. In the temperature range from 400 to 1000 °C, the M_23_C_6_ and M_6_C carbides were the main phases in the steel besides the matrix, and the dissolution of M_23_C_6_ carbide was completed at around 800 °C, while the dissolution of M_6_C was completed at about 850 °C. According to the calculation results, solid–solution treatment at temperatures above 850 °C can effectively dissolve carbides in the matrix. However, in practice, the equilibrium state is not reached in the heat treatment process, and if the solid–solution temperature is too high, the grain size will be too large, and the properties will degrade.

### 2.3. Mechanical Property Tests

Tensile and Charpy impact specimens were extracted from the mid–radius region along the longitudinal axis of the ingot and subjected to the heat treatment steps outlined in Figure 1. Tensile specimens were cylindrical (ϕ5 mm × 65 mm), while Charpy impact specimens were square–columnar (10 mm × 10 mm × 55 mm). Tensile tests were conducted on an MTS–880 universal testing machine (MTS–880, MTS Corporation, Woodbury, MN, USA) at a strain rate of 1 × 10^−2^ m/min under room temperature. Charpy impact tests were performed at ambient temperature using a JBN–300B impact tester. Two tensile specimens and two impact specimens were tested for each heat treatment condition to minimize data randomness. Microstructural samples were extracted via wire–cutting from the ends of tested impact specimens to ensure correlation between mechanical properties and microstructure in the experimental steel.

### 2.4. Microstructure Characterization

Microstructure observation specimens underwent sequential metallographic preparation beginning with mechanical grinding under controlled water–cooling conditions using silicon carbide papers of progressively finer grits (80#, 150#, 320#, 600#, 1000#, and 2000#), followed by diamond suspension polishing with 3 μm particles on napless polishing cloths at 150 rpm. Etching was performed through controlled swabbing with a freshly prepared 4 vol% nitric acid in ethanol solution for 5–10 s, immediately followed by water rinsing and oil–free compressed air drying to preserve boundary delineation. Microstructural characterization was conducted using a Zeiss Axio optical microscope (OM) under differential interference contrast illumination with 50 × −1000 × objectives, while simultaneous microstructure examination and carbide elemental characterization were performed via secondary electron detection at 15 kV acceleration voltage using a Quanta FEG 650 scanning electron microscope equipped with an energy–dispersive X–ray analysis (EDXA) detector. Quantitative characterization of prior austenite grains across various solid–solution temperatures was achieved through optimized image processing in Image–Pro Plus^®^ software (v6.0), employing equivalent circle diameter statistical analysis for specific measurements. Specimens designated for electron backscatter diffraction (EBSD) analysis received supplementary surface preparation through vibratory polishing with a 0.05 μm colloidal silica suspension on synthetic velvet cloths at 25 rpm for 6 h, followed by ultrasonic cleaning in deionized water for 10 min. EBSD mapping was executed across 40 μm × 40 μm areas with a 100 nm step size under 20 kV acceleration voltage and 8 nA probe current. Phase quantification was performed through pattern indexing using dynamically simulated reference crystals for austenite and martensite in AZTec software (version 2.1), which further enabled the extraction of martensite grain size parameters and geometrically necessary dislocation density data from specimens subjected to different thermal conditions. Transmission electron microscopy (TEM) analysis was performed using a Tecnai G2 20 instrument equipped with a LaB6 filament, operating at an accelerating voltage of 200 kV, including selected–area electron diffraction (SAED) for crystallographic characterization and energy–dispersive X–ray spectroscopy (EDS) for elemental composition mapping. TEM specimens were prepared by wire–cutting bulk materials into 0.3 mm–thick slices, mechanically ground to 40 μm thickness, and punched into 3 mm diameter discs. Electrolytic twin–jet polishing was subsequently conducted at −15 °C using a constant–current mode (40 mA) with a 6 vol% perchloric acid alcoholic solution as electrolyte for approximately 40 s.

## 3. Results and Discussion

### 3.1. Mechanical Properties and Fracture Appearance

**Figure 3:**
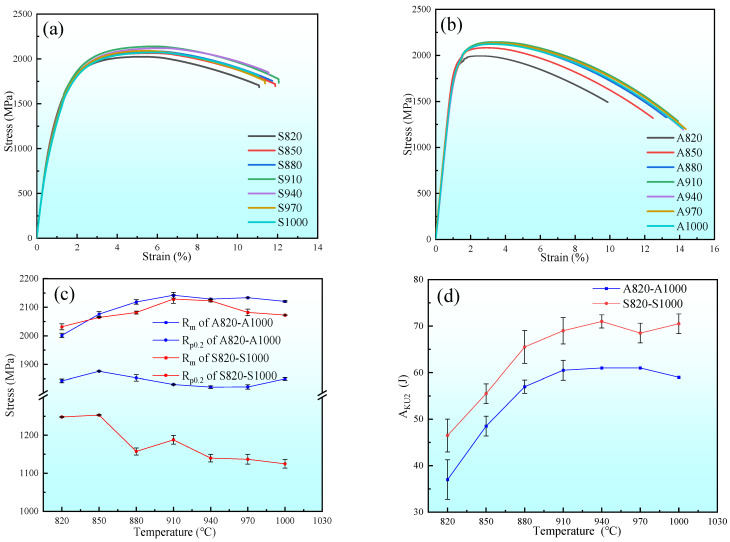
Mechanical properties of samples subjected to different heat treatment processes. (**a**) Stress–strain curves without aging at different solid–solution temperatures; (**b**) Stress–strain curves after aging at different solid–solution temperatures; (**c**) R_m_ and R_p0.2_ for different specimens; (**d**) A_KU2_ for different specimens.

Figure 3 shows the tensile and impact test results of all specimens at room temperature. In particular, the stress–strain curves shown in Figure 3a,b represent the unaged and aged states, respectively. Figure 3c,d, respectively, depict the solid–solution temperature dependence of tensile strength (R_m_), yield strength (R_p0.2_), and impact energy (A_KU2_). The R_m_ and R_p0.2_ of the specimens after aging are higher than those without aging. This phenomenon occurs due to the strengthening effect of nanoscale M_2_C precipitation after aging [28,29]. Although the Rm of these two different heat treatment processes first increases and then decreases with the increase in solid–solution temperature, reaching its maximum value at 910, the R_p0.2_ of the aged specimens first increases and then stabilizes, while the unaged specimens show a continuous downward trend.

As shown in Figure 3d, the impact energy of both aged and unaged specimens increased with rising solid–solution temperature, showing the most pronounced enhancement between 820 °C and 880 °C, and reaching a maximum at 940 °C. At 970 °C, a marginal toughness reduction was observed. Furthermore, the A_KU2_ value prior to aging exceeds that post–aging, attributable to the precipitation of M_2_C following the aging process [15]. Within the solid–solution temperature spectrum of 820 °C to 1000 °C, taking into account the dual considerations of strength–toughness balance and resource conservation, the optimal solid–solution temperature was determined to be 910 °C. At this temperature, the values for R_m_, R_p0.2_, and A_KU2_ were 2142 MPa, 1830 MPa, and 60.5 J, respectively.

**Figure 4:**
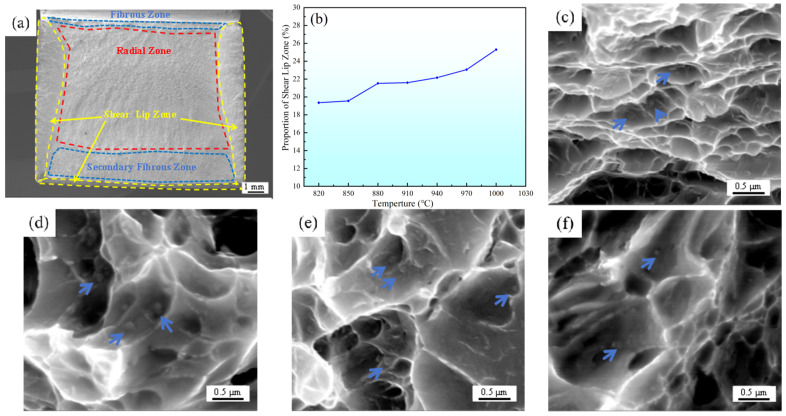
Impact fracture morphology of experimental steel treated by different solid–solution processes. (**a**) Macroscopic morphology of impact fracture surface; (**b**) The proportion of shear lip zone changes with temperature; (**c**) Microscopic impact fracture morphology of S820; (**d**) Microscopic impact fracture morphology of S850; (**e**) Microscopic impact fracture morphology of S880; (**f**) Microscopic impact fracture morphology of S910.

Figure 4 illustrates the impact fracture morphology of the different specimens of experimental steel. As evidenced by the macroscopic fracture morphology (Figure 4a), the fracture surface exhibits three distinct regions: fibrous zone, radial zone, and shear lip zone. Quantitative evaluation of the shear lip area fractions (Figure 4b) revealed a direct proportionality between solid–solution temperature and shear lip proportion, which aligns with variations in the material’s plasticity. High magnification SEM examination of impact fracture surfaces revealed abundant coarse undissolved primary carbides in specimens subjected to solid–solution treatment within the 820–910 °C temperature range. Notably, these carbides are predominantly located at the centers of the dimples, and their size decreases with increasing solid–solution temperature, as denoted by the blue arrows in Figure 4c–f.

### 3.2. Microstructures

**Figure 5:**
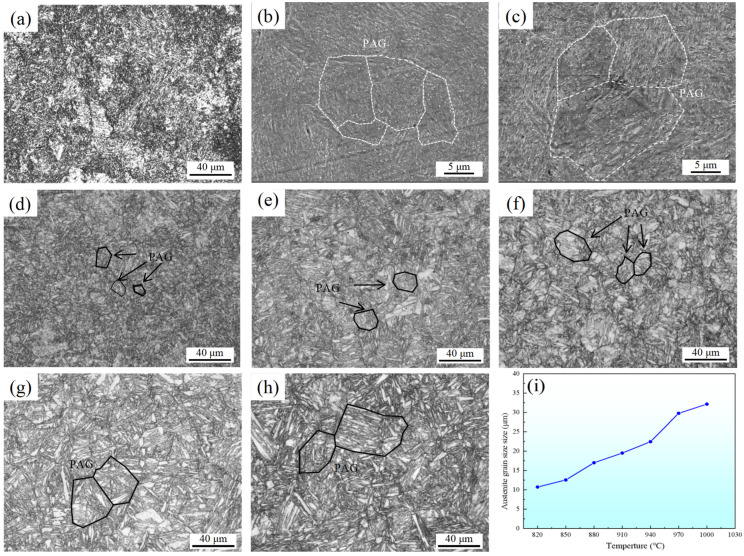
OM diagram of the unheated state and specimens subjected to different solid–solution treatment processes. (**a**) OM of original specimen; (**b**) SEM of A820; (**c**) SEM of A850; (**d**) OM of A880; (**e**) OM of A910; (**f**) OM of A940; (**g**) OM of A970; (**h**) OM of A1000; (**i**) The variation of PAG size with temperature.

**Figure 6:**
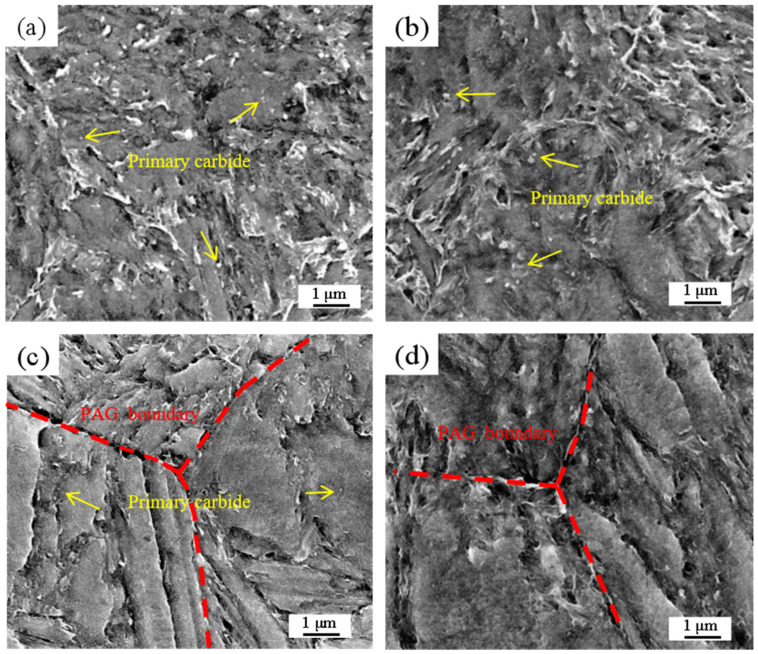
SEM diagrams of different solid–solution processes. (**a**) A850; (**b**) A880; (**c**) A910; (**d**) A970.

Figure 5 shows the OM and SEM images of different specimens. The figures reveal that the matrix structures resulting from solid–solution treatment are characterized by typical lath martensite. As the solid–solution temperature increases, the grain boundaries between different primary austenite grains (PAGs) become more pronounced, and the corresponding size of the PAG increases. The variation of the PAG size with solid–solution temperature is shown in Figure 5i. During the solid–solution temperatures of 820 °C and 850 °C, the PAG boundaries in the experimental steel exhibited incomplete growth and a lack of uniformity, yet the presence of numerous primary carbides was evident. At 880 °C, the complete prior austenite grain (PAG) boundary can be clearly observed under an optical microscope. This observation suggests that at this temperature, the dissolution of large–sized carbides weakens the grain boundary pinning effect, and the small grains are swallowed by the large grains due to their small radius of curvature (or high grain boundary curvature), resulting in a significant coarsening of the PAG [30,31]. And the size of primary carbides significantly decreases at this temperature, but these refined carbides are difficult to distinguish at the metallographic scale. Elevating the solid–solution temperature increases the grain boundary energy while the dissolution of primary carbides diminishes the resistance to grain boundary diffusion, thereby promoting the subsequent enlargement of the PAG dimensions and consequently resulting in the degradation of both strength and toughness in the experimental steel [32]. Figure 6. illustrates the SEM images of A850, A880, A910, and A970 specimens after corrosion with nitric acid alcohol solution. As indicated by the yellow arrow in Figure 6a, coarse primary carbides are densely and unevenly distributed within the test steel matrix. In the A880 specimen, most small–sized primary carbides are dissolved, while large–sized primary carbides persist. During solid–solution temperature at 910 °C, the primary carbides were nearly completely dissolved, while the primary austenite grains (PAGs) exhibited well–defined angular morphology and high microstructural uniformity, as demonstrated in Figure 6c. These experimental observations confirm that the temperature range for rapid dissolution of primary carbides occurs between 850 °C and 910 °C, which shows excellent agreement with both Thermo–Calc computational predictions and metallographic analysis results.

**Figure 7:**
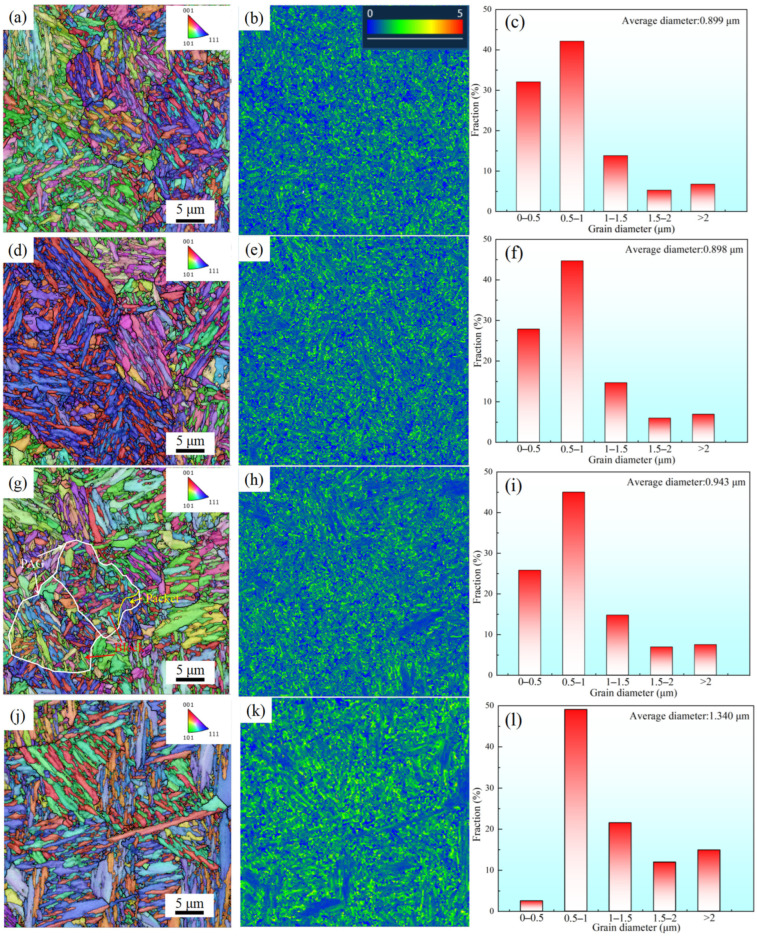
EBSD diagrams at different solid–solution temperatures. (**a**–**c**) IPF micrograph, KAM distribution, and martensitic grain size of A850; (**d**–**f**) IPF micrograph, KAM distribution, and martensitic grain size of A880; (**g**–**i**) IPF micrograph, KAM distribution, and martensitic grain size of A9100; (**j**–**l**) IPF micrograph, KAM distribution, and martensitic grain size of A970.

Figure 7 is the EBSD diagram of the specimens at 850 °C, 880 °C, 910 °C, and 970 °C. The PAG is subdivided into multiple packet structures (Packet), each composed of block subunits (Block) sharing identical crystallographic orientation relationships, as evidenced in Figure 7g. A significant grain coarsening phenomenon occurred in martensite with increasing solid–solution temperature. The average grain size was measured as 0.899 μm and 0.898 μm at 850 °C and 880 °C, respectively, while it increased to 0.943 μm and 1.34 μm when the temperature reached 910 °C and 1000 °C, indicating temperature–dependent coarsening behavior within the 880–1000 °C range.

Figure 7b,e,h,k displays the distribution of Kernel Average Misorientation (KAM, °) in EBSD for different samples using AZtec software. The color gradient from blue to red quantitatively reflects the spatial distribution density of KAM values, with blue regions indicating low local lattice distortion and red regions denoting high localized strain concentrations. KAM reflects the average orientation difference around the measurement point, which can be used to understand the lattice distortion inside the microstructure, thereby characterizing the geometrically necessary dislocation density (*ρ_GND_*) [33]. The *ρ_GND_* is dislocations that occur in crystalline materials during deformation due to deformation incompatibility near grain boundaries or phase boundaries.

The relationship between *ρ_GND_* and *KAM* can be calculated by the following formula:

(1) $$\rho_{GND}=\frac{2\times KAM_{ave}}{\mu b}$$

**Figure 8:**
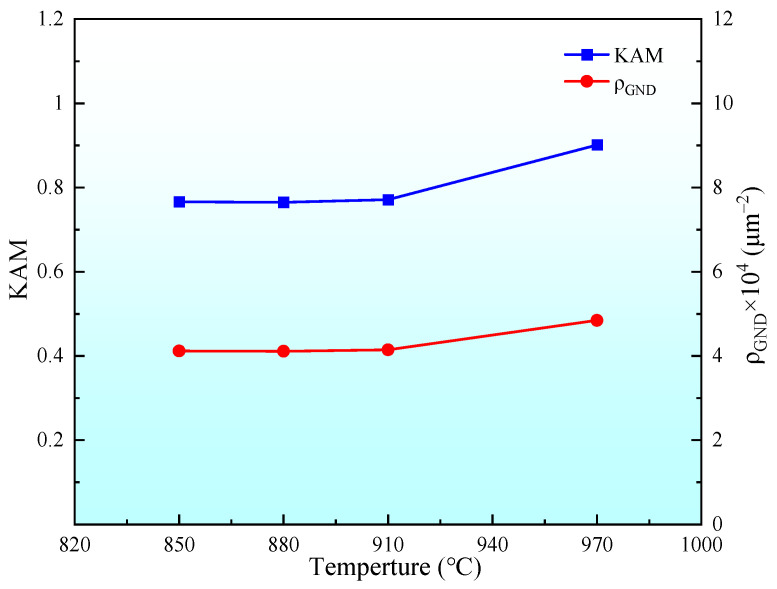
Changes of KAM and *ρ_GND_* with solid–solution temperature.

In the formula, *μ* is the EBSD scanning step, and *b* is the dislocation Burgers vector. In the experiment, *μ* = 150 nm, *b* = 0.248 nm. The average *KAM* values of specimens A850, A880, A910, and A970 were statistically analyzed using AZtec software, with the average *ρ_GND_* calculated via Equation (1). The dependence of both *KAM* and *ρ_GND_* on solid–solution temperature is illustrated in Figure 8. Martensitic transformation belongs to the shear process. The increase in solid–solution temperature increases the driving force for martensitic transformation, which leads to the increase in geometrically necessary dislocations [34].

### 3.3. The Influence of Solid–Solution Temperature on Undissolved Phases

**Figure 9:**
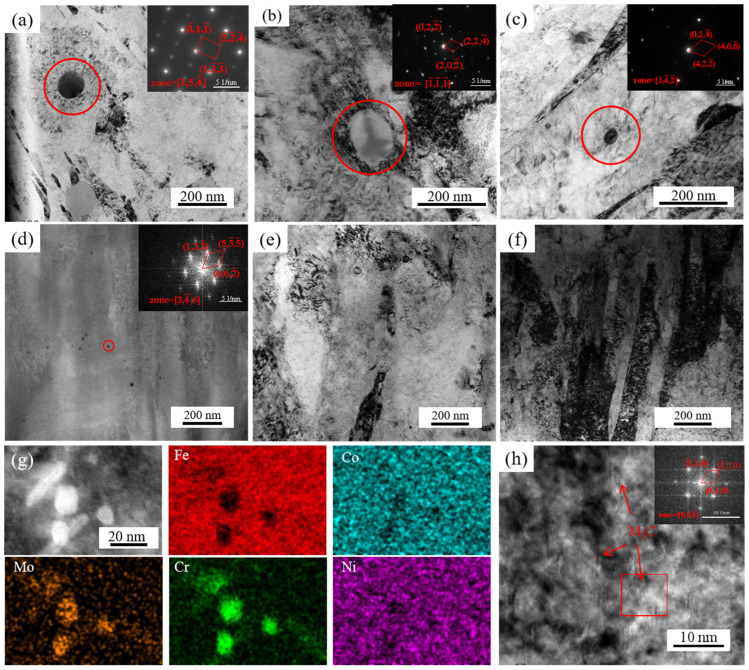
TEM micrographs and SAED patterns of different samples. (**a**–**f**) The morphology of primary carbides in (**a**–**h**) S820, S850, S880, S910, S940 and S970; (**g**) EDS results of primary carbides in S910; (**h**) M_2_C morphology of A910.

Figure 9 shows the TEM images and EDS results of the experimental steel. In the solid samples at low solid–solution temperatures (820–850 °C), a large amount of coarse primary carbides were observed in the steel, as illustrated in Figure 9a–c. The primary carbide size ranges from 70 to 120 nm at solid–solution temperatures of 820 °C and 850 °C. As the solid–solution temperature increases, the undissolved primary carbides progressively dissolve into the matrix, accompanied by a reduction in their dimensions. At 880 °C, the majority of the residual undissolved primary carbides exhibit sizes of approximately 50 nm, while a limited number of large–sized primary carbides (~100 nm) persist, which is detrimental to the mechanical properties of the steel. When the solid–solution temperature rises to 910 °C, the size of the residual primary carbides decreases to ~20 nm. Notably, the carbide size in specimens treated at 940 °C exhibits minimal variation compared to those at 910 °C, though their distribution density is significantly reduced. Fine–scale carbides are advantageous for enhancing steel properties. Despite the increased precipitation of M_2_C carbides at 940 °C relative to 910 °C, the coarser PAG size at elevated temperatures contributes to marginally reduced strength at 940 °C compared to 910 °C. Unlike the rapid coarsening of primary austenite grains (PAG) at 880 °C, at a solid–solution temperature of 970 °C, the primary carbides fully dissolve into the matrix, eliminating their pinning effect on grain boundary migration. Moreover, the higher solid–solution temperature results in substantial coarsening of PAGs at 970 °C. However, it simultaneously promotes M_2_C precipitation during aging. The synergistic effects of these mechanisms result in only minor degradation of the steel’s strength and toughness [24,26].

As shown in Figure 9, the undissolved primary carbides in the experimental steel were characterized using EDS analysis and SAED calibration. These carbides were identified as Mo– and Cr–rich M_6_C–type phases.

## 4. Conclusions

The strength–toughness synergy of the experimental steel, as an aerospace–grade structural material, constitutes the research focus. Optimization of solid–solution parameters serve as the critical precursor for achieving balanced strength–toughness combinations, providing fundamental guidance for industrial–scale processing. This study systematically investigates the temperature–dependent evolution of the mechanical properties and the microstructural characteristics during solid–solution treatment (820–1000 °C), identifies the optimal heat treatment protocol, and conducts a preliminary assessment of contributions from prior austenite grain structure, dislocation density, and primary carbide dissolution. The principal findings are summarized as follows:

(1) Within the temperature range of 820–1000 °C, both strength and toughness of the experimental steel exhibited an initial increase followed by a subsequent decrease with increasing solution temperature. At 910 °C, the dissolution of coarse carbides, combined with fine grain sizes of prior austenite and lath martensite, high dislocation density, and age–precipitated nano–sized M_2_C carbides, resulted in the optimal strength–toughness synergy in the steel.

(2) During solid–solution at 820–1000 °C, R_P0.2_ initially increases and then stabilizes, which can be attributed to the coarsening of PAGs, the increase in lath martensite size, the dissolution of M_6_C carbides, and the precipitation of M_2_C carbides. The dissolution of large–sized M_6_C carbides and the precipitation of M_2_C carbides promote the strength of the steel, while the coarsening of PAGs and lath martensite exerts a detrimental effect on the strength.

(3) Within the solid–solution temperature range of 820–940 °C, the initial increase in impact toughness is attributed to the dissolution of large–sized M_6_C carbides and improved microstructural homogenization, which inhibits crack propagation, thereby enhancing toughness. When the solid–solution temperature exceeds 940 °C, excessive coarsening of prior austenite grains and martensite blocks occurs, leading to a reduction in toughness.

## Figures and Tables

**Table 1:** Nominal chemical compositions of the experimental steel (mass %).

C	Ni	Mo	Cr	Co	Fe
0.25	11	1.5	2.3	15	Bal.

## Data Availability

All the data that support the findings of this study are available upon reasonable request.

## Abbreviations

Symbol	Significance
UHSS	Ultra–high–strength steels
HCP	Hexagonal close–packed
PAG	Primary austenite grain
*ρ_GND_*	Geometrically necessary dislocation density
HAGB	High angle grain boundaries
EDS	Energy–dispersive spectroscopy
SEAD	Selected area electron diffraction
SEM	Scanning electron microscope
TEM	Transmission electron microscopy
OM	Optical microscope
R_m_	Tensile strength
R_p0.2_	Yield strength
KAM	Kernel Average Misorientation
